# VisTCR: An Interactive Software for T Cell Repertoire Sequencing Data Analysis

**DOI:** 10.3389/fgene.2020.00771

**Published:** 2020-07-21

**Authors:** Qingshan Ni, Jianyang Zhang, Zihan Zheng, Gang Chen, Laura Christian, Juha Grönholm, Haili Yu, Daxue Zhou, Yuan Zhuang, Qi-Jing Li, Ying Wan

**Affiliations:** ^1^Biomedical Analysis Center, Army Medical University, Chongqing, China; ^2^Chongqing Key Laboratory of Cytomics, Chongqing, China; ^3^Biowavelet Ltd., Chongqing, China; ^4^Department of Immunology, Duke University Medical Center, Durham, NC, United States; ^5^Molecular Development of the Immune System Section, NIAID Clinical Genomics Program, National Institute of Allergy and Infectious Diseases, National Institutes of Health, Bethesda, MD, United States

**Keywords:** T cell sequencing, analysis tool, data analysis, Graphic user interface, T cell repertoire

## Abstract

Recent progress in high throughput sequencing technologies has provided an opportunity to probe T cell receptor (TCR) repertoire, bringing about an explosion of TCR sequencing data and analysis tools. For easier and more heuristic analysis TCR sequencing data, we developed a client-based HTML program (VisTCR). It has a data storage module and a data analysis module that integrate multiple cutting-edge analysis algorithms in a hierarchical fashion. Researchers can group and re-group samples for different analysis purposes by customized “Experiment Design File.” Moreover, the VisTCR provides a user-friendly interactive interface, by all the TCR analysis methods and visualization results can be accessed and saved as tables or graphs in the process of analysis. The source code is freely available at https://github.com/qingshanni/VisTCR.

## Introduction

Breakthroughs made in the development of antibody-based treatments for autoimmune diseases and tumor immunotherapy in recent have fueled an as-yet unmet need for feasible personal immune monitoring platforms to evaluate adaptive immune response (Han et al., 2015). T cells are one of the most critical players of adaptive immunity, with diverse functions including cell killing, providing B cell help (and consequently boost specific antibody production), and cytokine secretion. By capturing the identity and relative size of T cell clones, T cell receptor (TCR)-Seq offers an opportunity to observe changes in the composition of the adaptive immune system at homeostasis or during pathogenic responses (Aris et al., 2018; Fahl et al., 2018; Jiang et al., 2018). Sorting and clonotyping of purified T cell populations, such as Tregs, has yielded insight into pathogenic populations and phenotypic changes in autoimmunity, while the clarification of the clonal dynamics of tumor-infiltrating CD8^+^ T cells responsive to tumor neoantigens is under intensive study due to their positive association with enhanced prognosis. This additional dimension of immune monitoring thus extends our understanding of adaptive immunity, and has the potential to inform treatment decisions.

Facilitated in part by the decreasing cost of next-generation sequencing, T cell repertoire sequencing (TCR-Seq) data has been rapidly generated in recent years (Robins, 2013; Six et al., 2013; Newell and Davis, 2014; Hou et al., 2016). Many tools have also been developed for T cell sequencing data analysis. Some of these focus on sequence assembly, assignment to genomic V, D and J genes, extraction of CDR3 regions and error correction, such as IgBlast (Ye et al., 2013), TCRKlass (Yang et al., 2014), Decombinator (Thomas et al., 2013), IMSEQ (Kuchenbecker et al., 2015), MiTCR (Bolotin et al., 2013), and MiXCR (Bolotin et al., 2015). Others provide global evaluation methods on the TCR sequencing data, such as ARResT/Interrogate (Bystry et al., 2017), ImmunExplorer (Schaller et al., 2015), VDJtools (Gardner et al., 2015), VDJviz (Bagaev et al., 2016), Vidjil (Duez et al., 2016), and tcR (Nazarov et al., 2015), providing different methods to gain biological and clinical understanding by diversity measurements, clonotype distribution, similarity analysis, etc. Many of these tools also offer different types of visualizations for a given analysis that emphasize distinct interpretations. For instance, VDJviz can generate individual-sample circus plots for VJ usage, while tcR offers radar plots to emphasize divergence in VJ segments across samples. Other features, such as clonotype clustering in VDJil, may be more rarely provided by an individual tool.

However, these initial clonotype extraction and final visualization tools tend to be separated, and not all of these tools are readily intercompatible. As such, performing a more complete analysis of TCR repertoires would require a user to piece several of these tools together in order to generate comprehensive visualizations. Furthermore, most of the current tools are primarily operated by a command line interface, and data interpretation from such interfaces may be challenging for some wet lab immunological researchers, who may require extensive assistance from computational bioinformaticians to generate these analysis. The nuances between, and functional impact of applying, different clonotype extraction methods in terms of downstream interpretation may also be confusing. To overcome this barrier, we have developed the VisTCR (Visual TCRSeq) software, an interactive platform with a graphical user interface (GUI) for simplified management and analysis of TCR sequencing data. Starting from raw sequencing data, VisTCR can be used to directly perform clonotype extraction and downstream analyses within a single data management framework. VisTCR leverages three of the most commonly used extraction methods to allow users to more easily explore their data, and investigate the differences that may result from applying distinct analysis pipelines across a broad range of downstream visualizations.

## Design and Implementation

**FIGURE 1:**
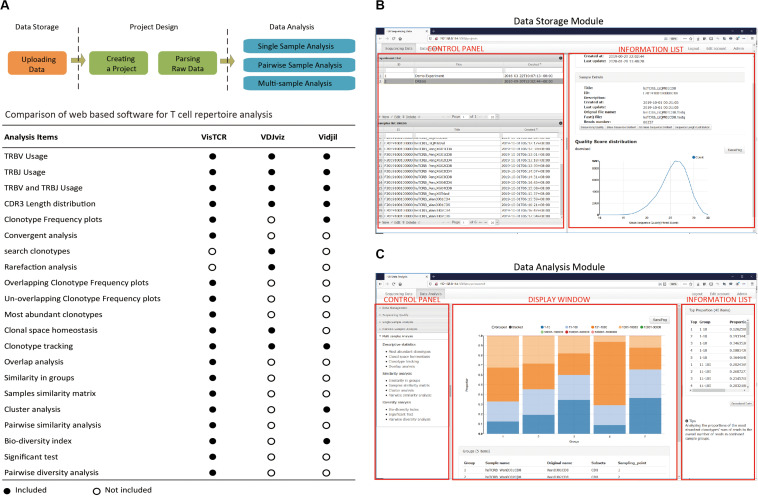
Overview of the VisTCR software. **(A)** Basic workflow of the VisTCR software and TCR sequencing data analysis methods in VisTCR software. The workflow of the VisTCR software includes three steps (1) Uploading TCR sequencing data files in the Data Storage Module, (2) Creating an analysis project in the Data Analysis Module. (3) Performing analysis and obtaining results in the Data Analysis Module. **(B)** The GUI of the Data Storage Module. **(C)** The GUI of the Data Analysis Module.

The design of VisTCR emphasizes a friendly, GUI and intuitive analysis workflow. The major features of the software include:

1. Independent modules for data management and analysis. In the Data Storage Module, raw data are uploaded and grouped in each sequencing experiments (Figure 1B and Supplementary Video S1). In the Data Analysis Module, the raw data can be selected and re-organized to perform various analyses and generate figures (Figure 1C and Supplementary Video S2).
2. Freedom to group samples for individualized analysis. An “Experiment Design File” is introduced in VisTCR that contains a combination of multiple variables for an analysis task, which allows users to de-construct their experiment data into a complex analysis design. Furthermore, in the data analysis process, individual variables or any combination of variables can be selected to group and re-group samples for comparison and analysis of T-cell sequencing data (Supplementary Files S1, S2 and Supplementary Video S2).
3. Integration of multiple cutting-edge analysis algorithms in a hierarchical fashion. These data analysis methods in VisTCR are organized in hierarchical fashion and are divided into three categories: Single sample analysis, Pairwise samples analysis, and Multi-samples analysis. Each category is further subdivided to generate comprehensive repertoire analysis that includes visualizing clonotype distribution, similarity analysis and diversity analysis, and tracking individual clones across samples, etc. (Figure 1A and Supplementary Table S1 and Supplementary Video S2).
4. User-friendly interactive interface and visualization of data. VisTCR provides a point and click interface for all of the TCR analysis methods. The analytical results are transformed into interactive data visualization with a representation-transparent approach (Bostock et al., 2011). These results can be downloaded as tables or graphs during each stage in the analysis workflow.

The workflow of VisTCR is composed of three steps (Figure 1A): (1) Uploading the sequencing data files into Data Storage Module, (2) Creating an analysis task in the Data Analysis Module, and (3) Performing analysis in Data Analysis Module. VisTCR use standard fastq format file as input, which is the most widely used format in sequence analysis. The raw TCR sequencing data files are uploaded, stored and organized in the “Experiment” tab of Data Storage Module (Figure 1B and Supplementary Video S1). A quality control tool (FastQC)^1^ has been integrated to Data Storage Module for assessment of sequencing quality (Supplementary Video S1). In Data Analysis Module, an “Experiment Design File” is created firstly with a list of samples and variables to import the raw data from Data Storage Module into analysis workflow (Supplementary Files S1, S2 and Supplementary Video S2). The raw TCR sequencing data can be parsed with several decoding methods [Decombinator (Thomas et al., 2013), MiTCR (Bolotin et al., 2013), and MiXCR (Bolotin et al., 2015)] as options (Supplementary Figure S1).

The analysis methods are categorized into three groups: Single sample analysis, Pairwise samples analysis, and Multi-samples analysis. In Single sample analysis, the TCRBV and/or TCRBJ usage, CDR3 spectratype and Clonotype distributions of selected samples can be analyzed. In Pairwise samples analysis, the shared clonotypes between two selected samples are shown in a plot with frequency of nucleotide or amino acid (nt/aa) sequences in Overlapping clonotype analysis. Moreover, the degeneracy of the shared T cell clonotypes is evaluated with Convergent Analysis, in which the number of unique CDR3 nucleotide sequences that are translated into same CDR3 amino acid sequence is calculated (Venturi et al., 2008). The Multi-sample analysis is classified into three categories: descriptive statistics, similarity analysis and diversity analysis. The description statistics contain Most Abundant Clonotypes, Clonal Space Homeostasis, Clonotype Tracking, and Overlap Analysis. The similarity analysis and diversity analysis provide statistical methods to quantify the differences of grouped datasets by using a variety of similarity and diversity estimation methods (Supplementary Table S1). A list of the analyses that are possible in VisTCR with respect to two other commonly used tools featuring GUIs is also included for ease of comparison (Figure 1A). Notably, VisTCR enables a number of unique analyses for sequence convergence and clonotype overlap that are not available in the other tools.

The software is a client-based HTML program that has an intuitive user interface which is written in ROR (Ruby on Rails) (Bachle and Kirchberg, 2007), and Data-driven documents Javascript library (D3.js) (Bostock et al., 2011). The calculation is implemented using R language, which is integrated with ROR using Rserve^2^.

## Results

To demonstrate the usage of VisTCR in T-cell repertoire analysis, a data set from a previously published paper was re-analyzed (Niu et al., 2015). As part of the original study to longitudinally characterize the CD4^+^/CD8^+^ T-cell repertoires in drug reaction with eosinophilia and systemic symptoms (DRESS) from diagnosis to clinical remission, CD4^+^ and CD8^+^ T-cells from peripheral blood of DRESS patients were isolated at 10-day intervals, and sequenced CDR3-regions of the TCRB chain on Ion Torrent PGM platform (Life Technologies, Carlsbad, CA, United States). This data set includes 66 samples from eight DRESS patient and 28 samples from healthy donors (Niu et al., 2015). All samples were uploaded into the data management module of VisTCR (Supplementary Video S1). Two experiment design files (Supplementary Files S1, S2) were edited to re-organize the data set. After uploading the experiment design files in the analysis module, two analysis tasks were created to demonstrate the cutting-edge analysis functions of VisTCR (Supplementary Video S2). One analysis task grouped the five timepoint TCR sequencing data from WDJ patient (Supplementary File S1). Another grouped the TCR sequencing data from the eight healthy donors together with samples taken at the first time pointfrom eight DRESS patients (Supplementary File S2). MiXCR with default parameters was used to extract CDR3 regions from raw sequences and perform error correction.

### Single Sample Analysis

**FIGURE 2:**
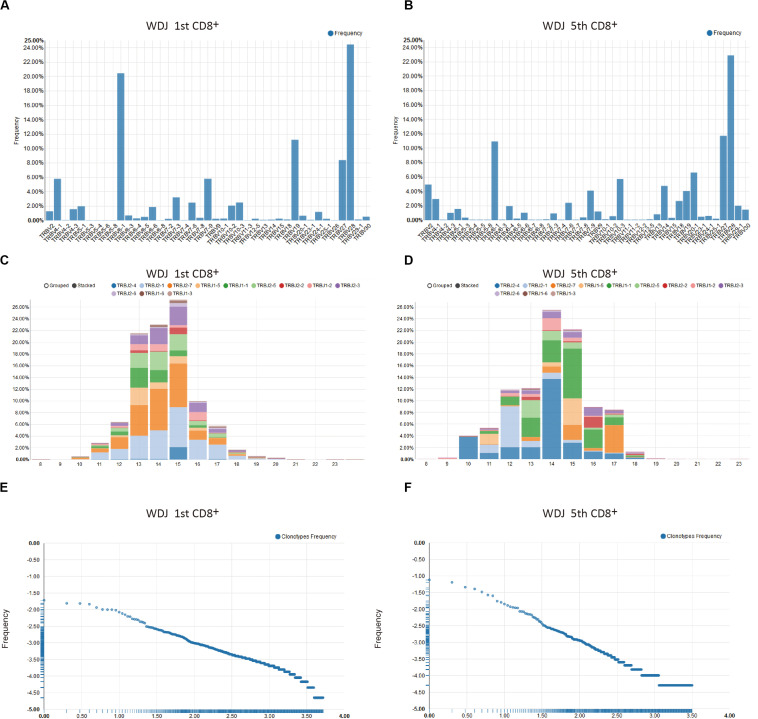
Examples of single sample analysis in VisTCR. The two samples are CD8^+^ repertoires and obtained at time point 1 and 5 from a representative patient are shown (patient WDJ). **(A,B)** TRBV segment usage. **(C,D)** The CDR3 length distribution with TRBJ segment usage. **(E,F)** Clonotype Frequency plots.

The Single Sample Analysis in VisTCR was provided to browse the fundamental characters of TCR sequencing data to uncover clues for further analysis of each given sample. For instance, significant differences between the first and fifth timepoint data for the samples from patient WDJ (an obscured patient ID) could be found in terms of TRBV/J segment usage, CDR3 length distribution, and clonotype distribution could be observed from this analysis (Supplementary Video S3 and Figure 2). The increase usage of TRBV27, TRBV13, TRBV18 and decreased usage of TRBV5-8, TRBV19 were discovered in the TRBV usages of the two timepoint data (Figures 2A,B). The peak of CDR3 length was 45 bp at the first timepoint and 42 bp by the fifth timepoint (Figures 2C,D). The highest frequency of TCR clonotype reached 10% in fifth timepoint, but had only reached 1.8% in first timepoint (Figures 2E,F). These resulting visualizations are thus consistent with the original conclusion that a portion of the CD8 + T cells were rapidly expanding in DRESS patients.

### Pairwise Sample Analysis

**FIGURE 3:**
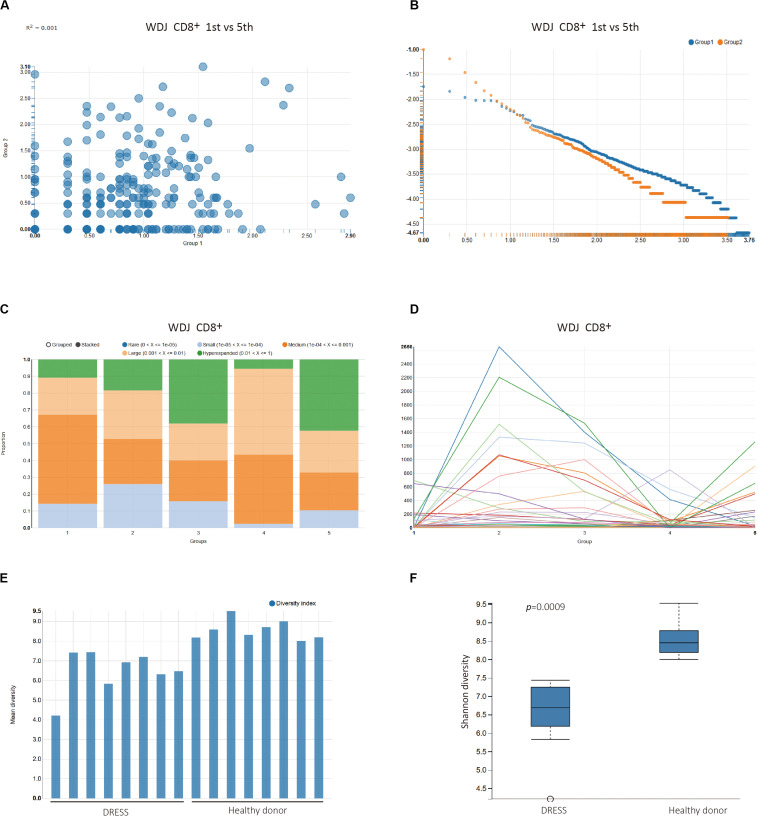
Multi-time point sample analysis of patient WDJ. **(A)** Overlapping Clonotype Frequency plots between time point 1 (Group1) and time point 5 (Group 2). **(B)** Un-overlapping Clonotype Frequency plots of time point 1 (Group1) and time point 5 (Group 2). **(C)** Clonal space homeostasis visualization to help identify potential clonal expansion. The proportional distribution of the fourth timepoint TCR clonotypes differed from the third timepoint (*p* < 0.0001, Chi-square test) and fifth timepoint (*p* < 0.0001, Chi-square test). **(D)** Clonal tracking mapping the dominance of a given clone across all samples. Each line corresponds to a unique TCRB clonotype. As a general trend, it can be seen that a number of clones undergo clear expansion at the earlier timepoint (time2) before subsequently contracting (time4), a behavior consistent with memory T cell formation following the end of antigen exposure. **(E)** Bar plot of Shannon diversity index. Two groups, DRESS patients and healthy donors, of repertoires are selected and analyzed. **(F)** Box plot of the two groups.

To inspect the change of the repertoire of CD8^+^ T cells in the development of DRESS, the first and fifth timepoint TCR sequencing data of WDJ patient were selected to analyze the distribution of overlapped and un-overlapped clonotype in the section of Pairwise sample analysis (Supplementary Video S4 and Figures 3A,B). In the Overlapping Clonotype Frequency scatter plots, the distribution of the shared clonotypes from the selected pair of timepoint datasets deviated significantly from the diagonal. The coefficient of determination was only 0.001 between the two timepoints (Figure 3A). Furthermore, a lot of high frequency clonotypes were found in the fifth timepoint TCR sequencing data of WDJ patient from the Un-Overlapping Clonotype Frequency scatter plots (Figure 3B). The differences between the pair of TCR sequencing data is useful as a comparison between extremes in this demonstration (since there are additional timepoints), but may just as readily serve as the primary analysis of interest in alternative study designs.

### Multi-Sample Analysis

The section of Multi-samples Analysis provides a number of statistical analysis methods that are categorized into Description Statistics of TCR clonotypes, Similarity Statistical analysis between grouped datasets, and Biodiversity Statistical analysis of grouped datasets. The Description Statistics of TCR clonotypes was executed with pre-defined experimental factors Time_point in the WDJ Experiment Design Files (Supplementary Video S5 and Figures 3C,D). In Clonal space homeostasis analysis, it was shown that the proportional distribution of the fourth timepoint TCR clonotypes differed from other timepoint (Figure 3C). In Clonotype Tracking analysis, the change of the high frequency TCR clonotypes from five timepoint demonstrated that the CD8^+^ T cells of WDJ patient were expanded in second timepoint and contracted in third and fourth timepoint, then expanded in fifth timepoint again (Figure 3D). However, these types of visualizations can also be easily applied to explore the flow of T cell clones between different tissues, and each group can also be readily reordered to help facilitate ease of comprehension.

The statistical analysis on the similarity index and diversity index of TCR sequencing dataset also is developed in the VisTCR. For instance, the Bio-diversity index analysis calculated the diversity index of the TCR sequencing data according to factors set in the Experiment Design File (Supplementary Video S6 and Figure 3E). In Pairwise Diversity Analysis, it was found that the diversity index (Shannon entropy) of DRESS patients was significantly lower than healthy donors (*p* < 0.005, Wilcoxon Test). The lower diversity of DRESS patients is consistent with the expected expansion of antigen specific CD8 + T cells (Supplementary Video S6 and Figure 3F).

### Applicability of visTCR on Mouse Data

To further demonstrate the easy and general applicability of VisTCR, we also provide an additional worked example using a publicly available mouse tumor TCRseq dataset with a distinct experimental design (Aoki et al., 2018). Simple visualization of clonal homeostasis and Shannon diversity in the peripheral blood, tumor, and draining lymph node samples yielded the expected result of the tumor samples having lowered diversity and more highly expanded clones (Supplementary Figure 2A). Pairwise analysis of the blood and lymph node samples was similarly consistent with the reported results, and offered a simple statistical test for significance (Supplementary Figures 2B–E). Additional clustering and correlation across the three sample types considered could also be easily performed in VisTCR. The frequency of the dominant clone in the tumor samples could also be readily recovered and traced across the other samples. Taken together, VisTCR make it easier for users to perform their standard and unique analysis tasks.

### Additional Human Data Analysis of Sezary Syndrome

As an additional test case of the consistency of the VisTCR data analyses, we further replicated our workflow on a published dataset of peripheral blood samples from patients with Sezary syndrome, a form of cutaneous T cell lymphoma (Ruggiero et al., 2015). Consistent with the published results, the patients with Sezary syndrome showed more limited usage of TRBV chains compared to healthy controls (Supplementary Figures 3A,B). We could also observe that the Sezary patients had hyperexpansion of a number of clonotypes, with spectratyping showing a sharp dropoff in the detection of smaller clones as compared to healthy controls (Supplementary Figure 3C-D). These samples had lower performance in diversity metrics as a consequence (Supplementary Figures 3D,E). Taken together, these results generated using our analysis tool are qualitatively consistent with those generated using other utilities. VisTCR may thus also be useful for quickly performing third-party data re-analysis.

## Conclusion

VisTCR has been developed to parse, evaluate, and statistically analyze the TCR repertoire data with a user-friendly GUI. The data management module provides simple functions to organize the TCR sequencing data, and the data analysis module integrates most of the popular methods for TCR repertoire analysis with an intuitive analysis workflow. We believe that VisTCR may help make TCR repertoire analysis more accessible to wet-lab scientists, and help unlock the full potential of TCRseq data.

## Data Availability Statement

The open source code of VisTCR is available for free public download at the GitHub repository: https://github.com/qingshanni/VisTCR.Publicly available datasets were analyzed in this study. These data can be found here: SRA (PRJNA611474 and PRJNA287162) and GEO (GSE115425).

## Ethics Statement

Ethical review and approval was not required for this study because this study only involved re-analysis of published and publicly available datasets that had been previously approved and does not require further review as per institutional requirements. Original approval for the datasets used can be found in the papers referenced for each datasets cited.

## Author Contributions

Q-JL and YW designed the study. QN, JZ, and ZZ wrote the software code and prepared the figures. GC, LC, JG, HY, DZ, and YZ tested the function of the software. QN, JZ, ZZ, Q-JL, and YW wrote the manuscript. All authors contributed to the article and approved the submitted version.

## Conflict of Interest

ZZ was employed by Biowavelet Ltd., Chongqing, China. The remaining authors declare that the research was conducted in the absence of any commercial or financial relationships that could be construed as a potential conflict of interest.

## Footnotes

1 http://www.bioinformatics.babraham.ac.uk/projects/fastqc/

2 http://www.rforge.net/Rserve/

## Supplementary Material

FIGURE S1 The GUI of clone extract methods used in VisTCR. Three major methods are included and can be chosen by the user as follows; **(A)** Decombinator **(B)** MiTCR **(C)** MiXCR.

FIGURE S2 Example analysis of mouse tumor dataset by using VisTCR. **(A)** Clonal homeostasis analysis. **(B)** Bio-diversity analysis by using Shannon diversity index. **(C–E)** Pairwise diversity analysis.

TABLE S1 TCR sequencing data analysis methods in VisTCR software.

FILE S1 Experiment design file for analyzing all 5 CD8^+^ samples from DRESS patient WDJ. The experiment design file is used to define the specific experimental conditions and any dependent variables or factors that can be used in TCR repertoire data analysis.

FILE S2 Experiment design file for analyzing samples from 8 DRESS patients and 8 healthy donors.

VIDEO S1 Uploading the sequencing data files into Data Storage Module. This video displays the experimental data management functions provided by Data Storage Module in VisTCR. Firstly, an experiment is created with title and description. Then, the raw TCR sequencing data belonging to the experiment are uploaded one by one. Finally, the quality of raw sequencing data is checked.

VIDEO S2 Creating an analysis task in the Data Analysis Module. Firstly, experiment design files are created by using Notepad ++, and saved in the CSV format. Then, a new analysis project is created by using wizard mode in VisTCR. In this process, the project title and description is set, the method for parsing raw TCR sequencing data is selected, and the experiment design file created previously is uploaded.

VIDEO S3 Single sample analysis in VisTCR. This video displays single sample analysis functions provided by Data Analysis Module in VisTCR, including their TRBV and/or TRBJ usage, CDR3 spectratype, and their clonotype distribution.

VIDEO S4 Pairwise sample analysis in VisTCR. This video displays pairwise sample analysis functions provided by Data Analysis Module in VisTCR, including samples selection, overlapping and un-overlapping clonotype distribution and convergence analyses.

VIDEO S5 Description statistics analysis in VisTCR. This video displays description statistics analysis functions provided by Data Analysis Module in VisTCR, including most abundant clonotypes, clonal space homeostasis, clonotype tracking, overlap analysis.

VIDEO S6 Multi-sample analysis of DRESS patients and healthy donors. This video displays some multi-sample analysis functions used to analyze DRESS patients and healthy donors, including most abundant clonotypes, clonal space homeostasis, bio-diversity index, and pairwise diversity analysis.

## References

[B1] Aoki H. Ueha S. Shichino S. Ogiwara H. Hashimoto S. Kakimi K. (2018). TCR repertoire analysis reveals mobilization of novel CD8+ T cell clones into the cancer-immunity cycle following Anti-CD4 antibody administration. *Front. Immunol.* 2018:3185. 10.3389/fimmu.2018.03185 30733724 PMC6353793

[B2] Aris M. Bravo A. I. Pampena M. B. Blanco P. A. Carri I. Koile D. (2018). Changes in the TCRbeta repertoire and tumor immune signature from a cutaneous melanoma patient immunized with the CSF-470 vaccine: a case report. *Front. Immunol.* 9:955. 10.3389/fimmu.2018.00955 29774030 PMC5944263

[B3] Bachle M. Kirchberg P. (2007). Ruby on rails. *IEEE Softw.* 24 105–108.

[B4] Bagaev D. V. Zvyagin I. V. Putintseva E. V. Izraelson M. Britanova O. V. Chudakov D. M. (2016). VDJviz: a versatile browser for immunogenomics data. *BMC Genomics* 17:453. 10.1186/s12864-016-2799-7 27297497 PMC4907000

[B5] Bolotin D. A. Poslavsky S. Mitrophanov I. Shugay M. Mamedov I. Z. Putintseva E. V. (2015). MiXCR: software for comprehensive adaptive immunity profiling. *Nat. Methods* 12 380–381. 10.1038/nmeth.3364 25924071

[B6] Bolotin D. A. Shugay M. Mamedov I. Z. Putintseva E. V. Turchaninova M. A. Zvyagin I. V. (2013). MiTCR: software for T-cell receptor sequencing data analysis. *Nat. Methods* 10 813–814. 10.1038/nmeth.2555 23892897

[B7] Bostock M. Ogievetsky V. Heer J. (2011). D^3^ data-driven documents. *IEEE Trans. Vis. Computer Graph.* 17 2301–2309. 10.1109/TVCG.2011.185 22034350

[B8] Bystry V. Reigl T. Krejci A. Demko M. Hanakova B. Grioni A. (2017). ARResT/Interrogate: an interactive immunoprofiler for IG/TR NGS data. *Bioinformatics* 33 435–437. 10.1093/bioinformatics/btw634 28172348

[B9] Duez M. Giraud M. Herbert R. Rocher T. Salson M. Thonier F. (2016). Vidjil: a web platform for analysis of high-throughput repertoire sequencing. *PLoS One* 11:e0166126. 10.1371/journal.pone.0166126 27835690 PMC5106020

[B10] Fahl S. P. Coffey F. Kain L. Zarin P. Dunbrack R. L. Jr. Teyton L. (2018). Role of a selecting ligand in shaping the murine gammadelta-TCR repertoire. *Proc. Natl. Acad. Sci. U.S.A.* 115 1889–1894. 10.1073/pnas.1718328115 29432160 PMC5828614

[B11] Gardner P. P. Shugay M. Bagaev D. V. Turchaninova M. A. Bolotin D. A. Britanova O. V. (2015). VDJtools: unifying post-analysis of T cell receptor repertoires. *PLoS Comput. Biol.* 11:e1004503. 10.1371/journal.pcbi.1004503 26606115 PMC4659587

[B12] Han Y. Li H. Guan Y. Huang J. (2015). Immune repertoire: a potential biomarker and therapeutic for hepatocellular carcinoma. *Cancer Lett.* 379 206–212. 10.1016/j.canlet.2015.06.022 26188280

[B13] Hou X. L. Wang L. Ding Y. L. Xie Q. Diao H. Y. (2016). Current status and recent advances of next generation sequencing techniques in immunological repertoire. *Genes Immun.* 17 153–164. 10.1038/gene.2016.9 26963138

[B14] Jiang Q. Zhao T. Zheng W. Zhou J. Wang H. Dong H. (2018). Patient-shared TCRbeta-CDR3 clonotypes correlate with favorable prognosis in chronic hepatitis B. *Eur. J. Immunol*. 48 1539–1549. 10.1002/eji.201747327 29856484

[B15] Kuchenbecker L. Nienen M. Hecht J. Neumann A. U. Babel N. Reinert K. (2015). IMSEQ - a fast and error aware approach to immunogenetic sequence analysis. *Bioinformatics* 31 2963–2971. 10.1093/bioinformatics/btv309 25987567

[B16] Nazarov V. I. Pogorelyy M. V. Komech E. A. Zvyagin I. V. Bolotin D. A. Shugay M. (2015). tcR: an R package for T cell receptor repertoire advanced data analysis. *BMC Bioinformatics* 16:175. 10.1186/s12859-015-0613-1 26017500 PMC4445501

[B17] Newell E. W. Davis M. M. (2014). Beyond model antigens: high-dimensional methods for the analysis of antigen-specific T cells. *Nat. Biotechnol.* 32 149–157. 10.1038/nbt.2783 24441473 PMC4001742

[B18] Niu J. Jia Q. Ni Q. Yang Y. Chen G. Yang X. (2015). Association of CD8(+) T lymphocyte repertoire spreading with the severity of DRESS syndrome. *Sci. Rep.* 5:9913. 10.1038/srep09913 25905582 PMC4649994

[B19] Robins H. (2013). Immunosequencing: applications of immune repertoire deep sequencing. *Curr. Opin. Immunol.* 25 646–652. 10.1016/j.coi.2013.09.017 24140071

[B20] Ruggiero E. Nicolay J. P. Fronza R. Arens A. Paruzynski A. Nowrouzi A. (2015). High-resolution analysis of the human T-cell receptor repertoire. *Nat. Commun.* 6:8081. 10.1038/ncomms9081 26324409 PMC4569693

[B21] Schaller S. Weinberger J. Jimenez-Heredia R. Danzer M. Oberbauer R. Gabriel C. (2015). ImmunExplorer (IMEX): a software framework for diversity and clonality analyses of immunoglobulins and T cell receptors on the basis of IMGT/HighV-QUEST preprocessed NGS data. *BMC Bioinformatics* 16:252. 10.1186/s12859-015-0687-9 26264428 PMC4531494

[B22] Six A. Mariotti-Ferrandiz M. E. Chaara W. Magadan S. Pham H. P. Lefranc M. P. (2013). The past, present, and future of immune repertoire biology - the rise of next-generation repertoire analysis. *Front. Immunol.* 4:413. 10.3389/fimmu.2013.00413 24348479 PMC3841818

[B23] Thomas N. Heather J. Ndifon W. Shawe-Taylor J. Chain B. (2013). Decombinator: a tool for fast, efficient gene assignment in T-cell receptor sequences using a finite state machine. *Bioinformatics* 29 542–550. 10.1093/bioinformatics/btt004 23303508

[B25] Venturi V. Price D. A. Douek D. C. Davenport M. P. (2008). The molecular basis for public T-cell responses? *Nat. Rev. Immunol.* 8 231–238. 10.1038/nri2260 18301425

[B26] Yang X. Liu D. Lv N. Zhao F. Liu F. Zou J. (2014). TCRklass: a new K-string-based algorithm for human and mouse TCR repertoire characterization. *J. Immunol.* 194 446–454. 10.4049/jimmunol.1400711 25404364

[B27] Ye J. Ma N. Madden T. L. Ostell J. M. (2013). IgBLAST: an immunoglobulin variable domain sequence analysis tool. *Nucleic Acids Res.* 41 W34–W40. 10.1093/nar/gkt382 23671333 PMC3692102

